# Kinetics of mean platelet volume predicts mortality in patients with septic shock

**DOI:** 10.1371/journal.pone.0223553

**Published:** 2019-10-17

**Authors:** Fanny Vardon-Bounes, Marie-Pierre Gratacap, Samuel Groyer, Stéphanie Ruiz, Bernard Georges, Thierry Seguin, Cédric Garcia, Bernard Payrastre, Jean-Marie Conil, Vincent Minville

**Affiliations:** 1 Anesthesiology and Critical Care Unit, Toulouse University Hospital, Toulouse, France; 2 INSERM UMR 1048, Institut des Maladies Métaboliques et Cardiovasculaires, Equipe 11, Toulouse, France; 3 Hematology laboratory, Toulouse University Hospital, Toulouse, France; Vita Salute University of Milan, ITALY

## Abstract

**Introduction:**

Thrombocytopenia is well recognized as a poor prognosis sign associated with increased mortality and prolonged Intensive Care Unit (ICU) stay, particularly in septic patients. Mean platelet volume (MPV) could represent a relevant predictive marker of mortality. Here we investigated whether MPV kinetics during the first 15 days after hospital admission has a potential prognostic value for clinical outcome in septic shock.

**Methods:**

We performed a retrospectively analysis of a cohort of 301 septic patients admitted in ICU. Three-month mortality was the primary endpoint. The prognostic value of the covariates of interest was ascertained by multidimensional analysis. We proposed a classification and regression trees analysis to predict survival probability.

**Results:**

MPV kinetics was significantly different between 90-day survivors and non-survivors when followed during 15 days (except on day 3). 10-day MPV >11.6fL was an independent predictive factor of 90-day mortality (Hazard Ratio (HR) 3.796, 95% Confidence Interval (CI) [1.96–7.35], p = 0.0001) in multivariate analysis. Base excess on day 4 <1.9mmol/L was also a predictive factor of mortality (HR 2.972, 95%CI [1.38–6.40], p = 0.0054.

**Conclusion:**

MPV increase during the first 15 days after ICU admission in non-survivors was observed during septic shock and 10-day MPV >11.6fL was an independent predictive factor of 90-day mortality. This could be explained by the emergent response to acute platelet loss during septic shock, leading to megakaryocyte rupture to produce new but potentially immature platelets in the circulation. Therefore, continuous monitoring of MPV may be a useful parameter to stratify mortality risk in septic shock.

## Introduction

Sepsis has recently been defined as a “life-threatening organ dysfunction caused by a dysregulated host response to infection” [1]. Septic shock constitutes a “subset of sepsis in which both circulatory and cellular metabolism abnormalities occur”. Sepsis-related mortality is linked to multiple organ failure (MOF) development. MOF is partly due to microvascular thrombosis and endothelial dysfunction, involving thrombocytes. Thrombocytopenia is the most common hematologic disorder in the Intensive Care Unit (ICU) with a prevalence of around 50% [2]. Thrombocytopenia is well recognized as a poor prognosis sign and is associated with increased mortality and with a prolonged ICU stay [3–6]. Mean platelet volume (MPV) describes the average size of platelets in a blood sample. Many physicians have recently shown interest in MPV in several human studies in term of prognosis on short periods (one or two days) [7–10]. A mechanism that could explain the changes in MPV values is an adapted response to acute platelet loss during an inflammatory condition. Indeed, in physiological conditions, platelet count and thrombopoiesis from bone marrow megakaryocytes (MK) are tightly inter-regulated processes. In the presence of thrombopoietin (TPO), MK exhibit microtubule-dependent extensions of elongated pseudopodal structures called proplatelets allowing the release of newly generated platelets in the blood stream [9,11,12]. It is well known that younger platelets have a higher MPV. However, using intravital microscopy, Nishimura et *al*. have suggested that this process may not be sufficient to support a rapid platelet turnover, especially when the platelet need is acute [13]. Their team highlighted a mechanism for the rapid production of platelets and their release into the bloodstream thanks to a MK rupture process which leads to the rapid fragmentation of cytoplasmic prolongations. This leads to the release of a large number of thrombocytes into circulation. These platelets exhibit an increase in MPV and their morphology is somewhat different. This mode of release of young platelets from bone marrow megakaryocytes would restore a pool of circulating platelets in acute consumption situations such as sepsis. However, these platelets may have important functional differences due to a lesser organization of their microtubules and could therefore contribute to a poor clinical impact in septic patients. Little is known about the potential influence of MPV changes on mortality in a homogenous group of septic patients. Therefore, we focused our study on the MPV kinetics during the first 15 days after hospital admission to check if this parameter has a prognostic value for clinical outcome in septic shock.

## Materials and methods

### Patients and study design

This retrospective cohort study included patients with microbiologically proven septic shock who were admitted to the Intensive Care Unit (ICU) of a tertiary-care teaching hospital between January 2012 and January 2016. Patients received early gold-directed therapy (EGDT) as recommended.

Patients were excluded if they met these criteria: age <16, hemorrhagic shock, hemorrhagic surgery, patient under extracorporeal life support (ECLS), cardiac arrest, platelet transfusion and pre-existing thrombocytopenia or thrombocytopenia induced by chemotherapy.

This study was reviewed and approved by the institutional review board of Toulouse University Teaching Hospital, France (n°09–0916). All data were fully anonymized before we accessed them. No consent was necessary for this retrospective study.

The primary end-point was 90-day mortality.

### Data collection

We collected baseline characteristics including demographic information, neurologic and hemodynamic factors, laboratory data, duration of mechanical ventilation, renal replacement therapy, stay in ICU and clinical outcome 28 days after ICU admission, at 3 months (e.g. 90 days) and intrahospital mortality. For disease severity assessment, both new Simplified Acute Physiology Score (SAPS II) and Sequential Organ Failure Assessment (SOFA) were determined according to the worst values within the initial 24 hours of ICU admission.

Platelet count, serum mean platelet volume, hemoglobin (Hb), white blood cell (WBC) count, lactic acid, pH and base excess were measured during the 15 days after admission. Venous blood samples for laboratory counts were collected from all patients in tubes containing ethylenediamine tetra-acetic acid (EDTA) and analyzed with an SYSMEX XN 1000 hematology analyzer (Canada) within 30 minutes of sample collection. The normal reference range for MPV in our laboratory hospital is 8.5 to 12.2fL. Thrombocytopenia was defined by platelet count <150 G/L.). Day -1 corresponded to the day before the onset of sepsis for the patients already in hospital for another reason such as elective surgery.

### Statistical analysis

Continuous variables are expressed as median, 95% confidence interval (95% CI) and extreme values and categorical variables as numbers with percentages. Patients who died within 90 days after ICU admission were defined as “non-survivors”. Baseline characteristics are presented according to the occurrence of the primary outcome (survivors *vs*. non-survivors) and were compared between the 2 groups. We compared continuous variables using Mann-Whitney test and categorical variables using χ^2^ tests or Fisher exact tests. Correlations between the quantitative variables were realized by the Spearman Rank method.

Considering time-to-event, we constructed time-dependent receiver operating characteristics (ROC) curves to assess threshold and predictive values of the covariates of interest. Kaplan-Meier survival curves were produced using 90-day mortality based on MPV threshold value.

Covariate selection for the multivariate analysis was based on *P* value <0.2 in univariate analysis. The prognostic value of the covariates of interest was ascertained by Cox proportional hazards model, using covariates whose AUC contained 0.8 in the 95% confidence interval and the results are presented as hazard ratios (HR) with 95% CI. To highlight patients with the best survival chances, a partitioning of the population was represented using a Classification and Regression Trees (CART) analysis. The advantage of this approach is to describe the means of distribution of the population in homogeneous groups according to 90-day survival and the covariates selected from the multidimensional analysis[14].

Statistical analyses were conducted using SPSS® for Window version 23.0 (IBM Corporation, Chicago, IL). A *P* value <0.05 was considered statistically significant.

## Results

### Baseline characteristics

**Fig 1:**
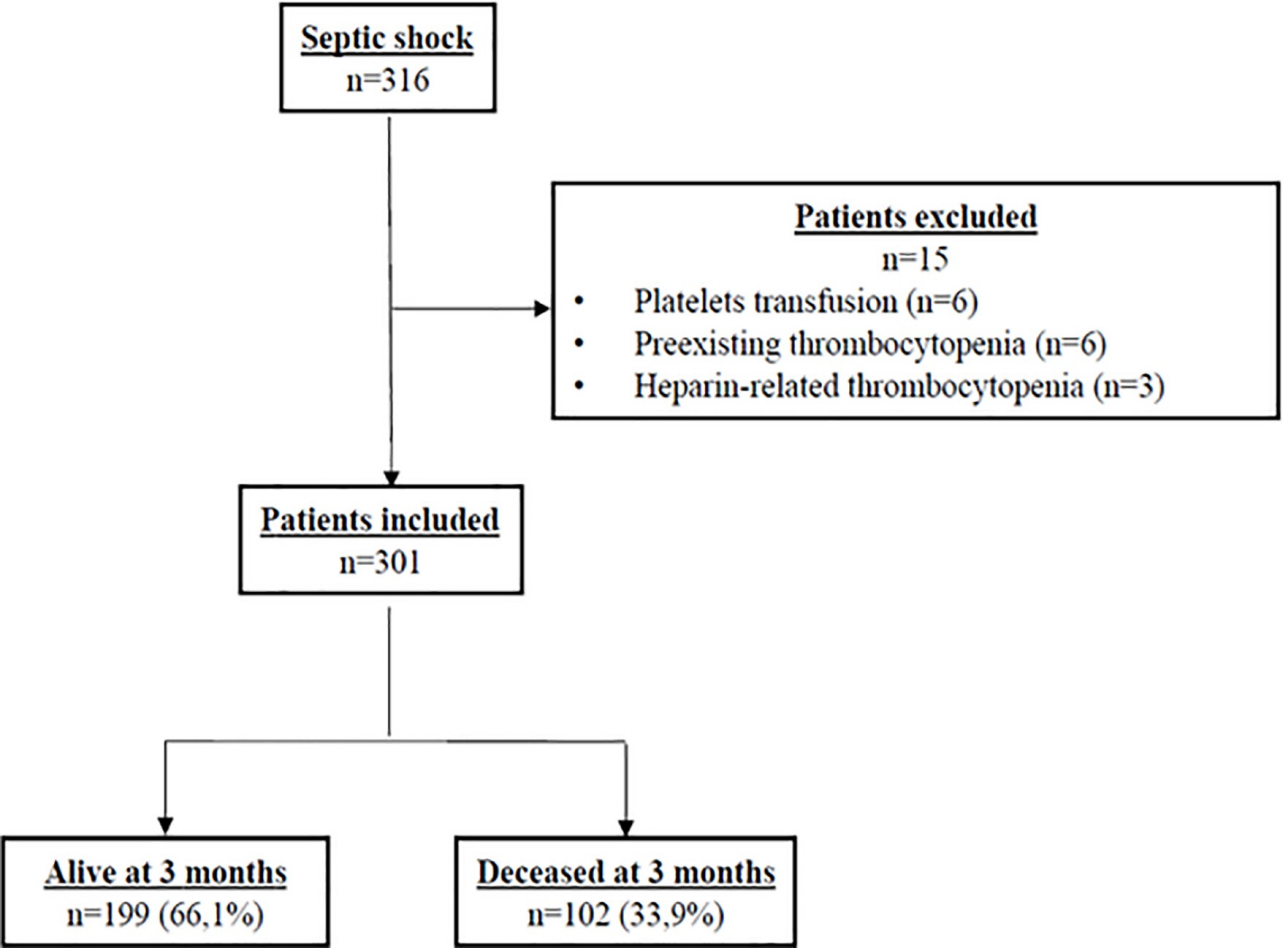
Flowchart of the enrolment of the patients in the study.

As shown in **Fig 1** illustrating the flow chart of the enrollment of patients used in our study, of the 316 consecutive patients admitted to the ICU, 15 were excluded. Among the 301 patients included, 102 (33.9%) were deceased at 3 months. The baseline demographic, clinical, and biological data of each group stratified by 90-day all-cause mortality are presented in **Table 1**. The main infection sites were lung (45.5%), and intra-abdominal cavity (29.2%), followed by urinary tract (20.9%). As shown in Table 1, 90-day survivors were younger than 90-day non-survivors. As expected, SOFA and SAPS II scores were higher in non-survivors. At admission, platelet count was not different between the 2 groups. However, MPV value, serum creatinine, total serum bilirubin and lactic acid were higher in non-survivors. The proportions of patients who received renal replacement therapy (RRT) and who required mechanical ventilation were significantly higher in non-survivors.

**Table 1:** Characteristics of the patients.

	Total Population (n = 301)	90-day survivors (n = 199)	90-day non survivors (n = 102)	*P*
**Demographic Data**				
Age (years)	66 (64–69)	64 (62–68)	71.5 (66–74)	0.0002***
Male sex. n (%)	197 (65.4)	130 (65.3)	67 (65.7)	
SOFA	11 (11–12)	10 (9–11)	14 (13–15)	< 0.0001***
SAPS II score	57 (52–60)	52 (48–56)	69 (61–76)	< 0.0001***
Antiplatelet therapy: yes (%)	34.6	32.8	38	ns
Time between onset of sepsis and ICU admission(D)	2 (2–2)	2 (2–2)	2 (2–2)	ns
Lenght of stay in ICU (D)	11 (10–13)	11 (10–14)	9 (4–12.4)	0.0044**
**Biochemical data at admission**				
Platelet count (G/L)	195 (187–218)	195 (181–219)	193 (178–239)	ns
MPV (fL)	10.5 (10.4–10.7)	10.4 (10.2–10.6)	10.8 (10.5–11.1)	0.0035**
Leukocyte count (G/L)	12.68 (11.26–14.069)	12.7 (10.9–14)	12.6 (11–15)	ns
Serum creatinine (μmol/L)	127 (119–140)	115 (99.6–127)	148 (129.6–198)	< 0.0001***
Total bilirubin (mmol/L)	11 (10–12.9)	10 (9.3–12)	13.5 (11–19)	0.0183*
Lactic acid (mmol/L)	2.6 (2.26–3.04)	2.3 (2–2.78)	3.3 (2.44–3.99)	0.0009***
pH	7.33 (7.31–7.35)	7.35 (7.32–7.37)	7.28 (7.25–7.33)	0.0022**
Base Excess (mmol/L)	-5.7 (-6.5; -4.90)	-5.1 (-6.13; -4.16)	-6.85 (-9.54; -5.56)	0.011*
Day of platelet count nadir (D)	3 (3–3)	3 (3–4)	3 (2–4)	ns
Number of days of thrombocytopenia (D)	2 (1–3)	1 (1–3)	2 (1–3)	ns
**Infection site. n (%)**				
Lung	137 (45.5)	88 (44.2)	49 (48)	0.0018**
Intra-abdominal site	88 (29.2)	48 (24.1)	40 (39.2)	
Urinary tract	63 (20.9)	52 (26.1)	11 (10.8)	
Bacteremia	13 (4.3)	11 (5.5)	2 (2)	
Digestive tract	88 (29.2)	48 (24.1)	40 (39.2)	0.00754**
**Mechanical Ventilation n (%)**	245 (81.4)	150 (75.4)	95 (93.1)	< 0.0001***
**Acute kidney injury n (%)**	209 (69.3)	120 (60.1)	89 (87.3)	< 0.0001***
**Renal Replacement Therapy n (%)**	84 (28)	37 (18.7)	47 (46.1)	< 0.0001***
**Vasopressor use n (%)**	276 (91.7%)	177 (88.9%)	99 (97.1%)	0.015*

Data are median (CI 95%) or n (%). SOFA: Sequential Organ Failure Assessment; SAPS II: Simplified Acute Physiology Score Index 2; MPV: Mean Platelet Volume

*p<0.05

**p<0.01

***p<0.001

ns: non-significant.

### Kinetics of MPV during the first 15 days in ICU

**Fig 2:**
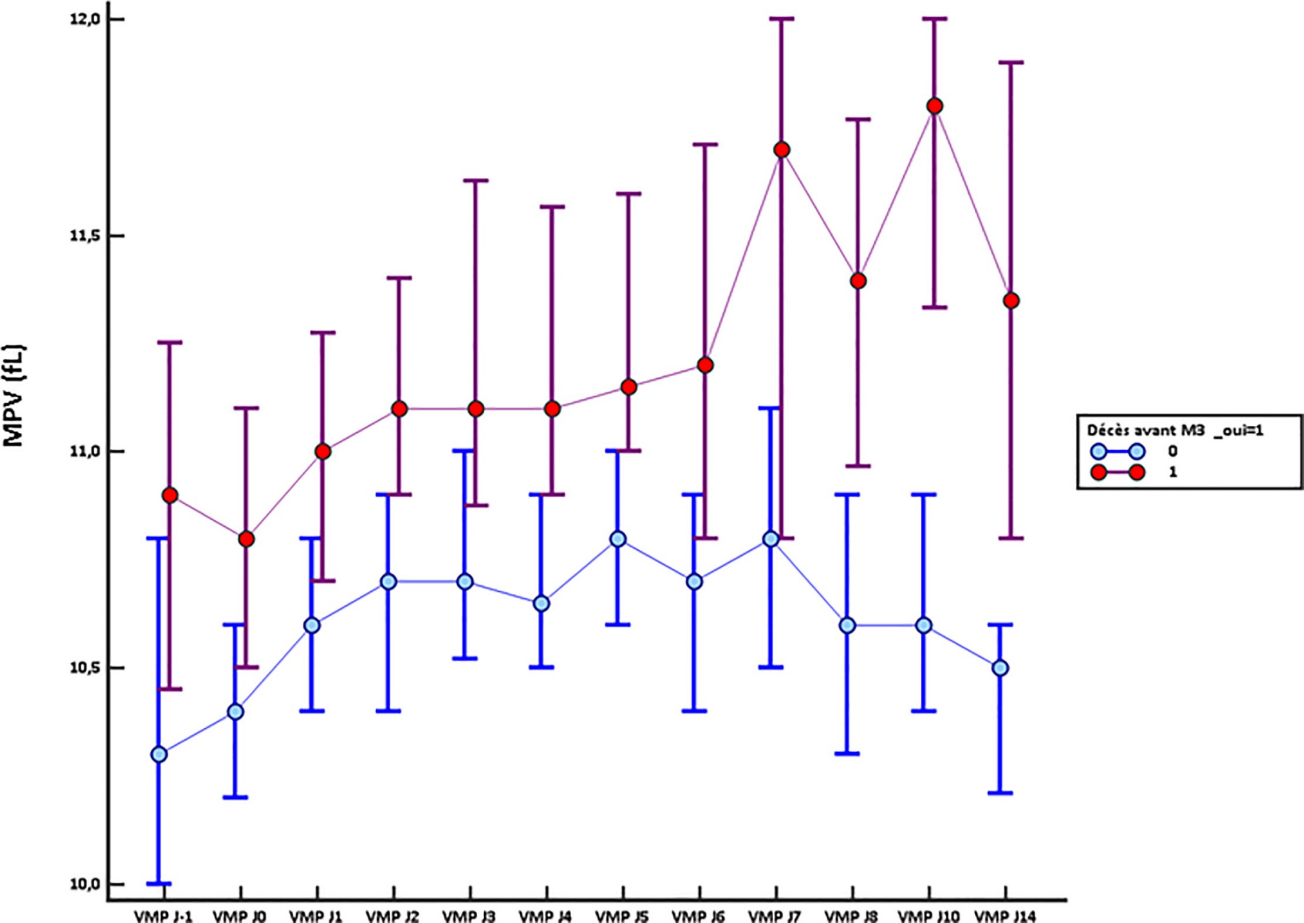
Comparison of MPV kinetics during 15 days according to 90-day survival.

Monitoring the MPV during the 15 days following admission in ICU indicated a significant difference between 90-day survivors and non-survivors since their admission for sepsis (10.4fL 95%CI [10.2–10.6] *vs* 10.8fL 95% CI [10.5–11.1] (p = 0.035) (**Fig 2).** Strikingly, the non-survivors exhibited a higher MPV all along the kinetics. Except for day 3, this difference was significant for all time points and was particularly clear after day 7. In the survivors group, MPV values were stabilizing as early as day 2 then declining after 7 days in ICU. MPV value was inversely significantly correlated with the value of the platelet count at each time (**S1 Table**). MPV was also significantly correlated with SOFA score at each time (**S2 Table**).

### Kinetics of platelet count during the first 15 days in ICU

**Fig 3:**
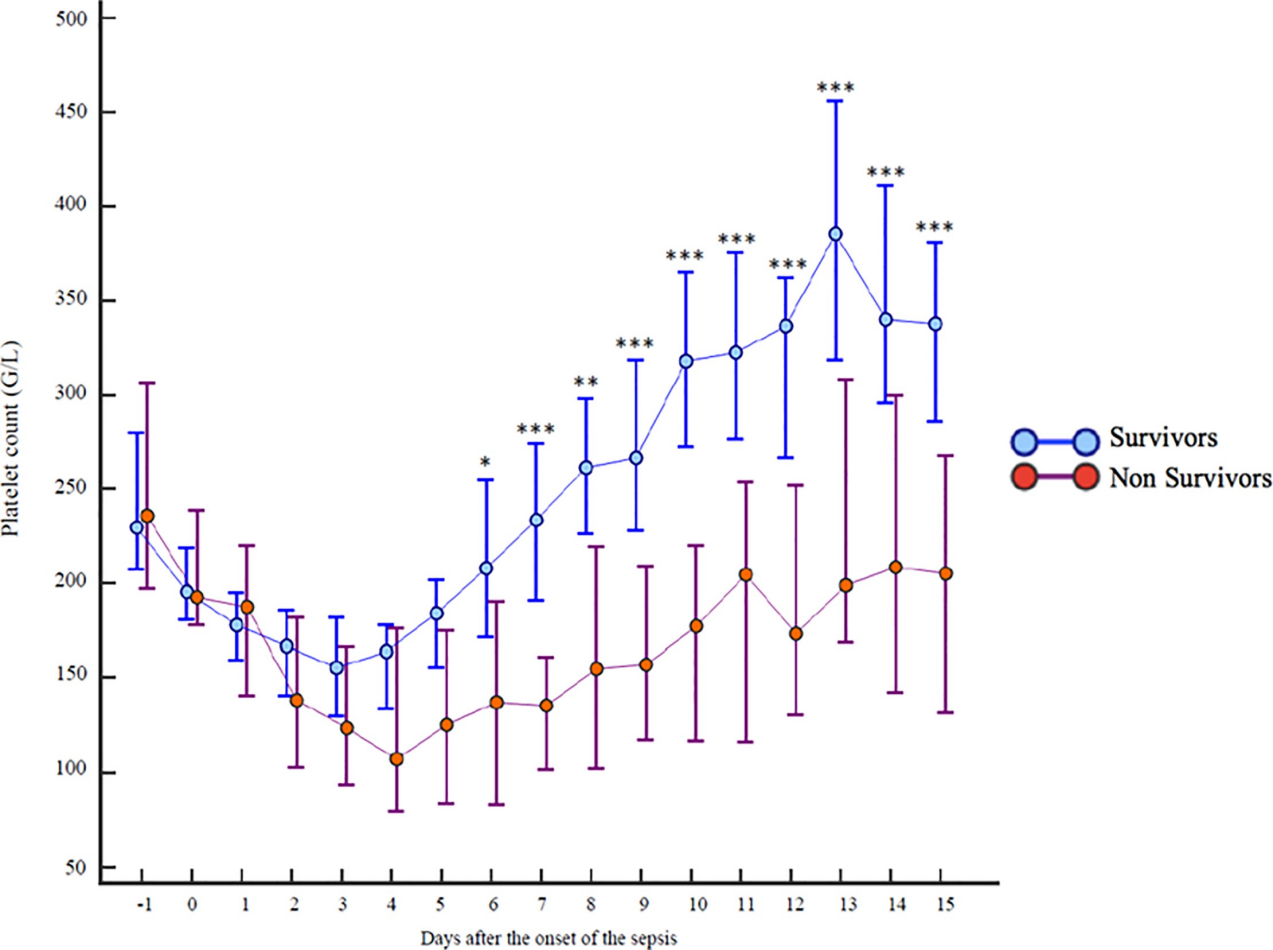
Comparison of platelet counts during 15 days according to 90-day survival.

Platelet count decreased significantly during the first days of ICU admission to reach a nadir on day 3 in the survivors group and on day 4 in the non-survivors group (**Fig 3**). The difference between the two groups was significant from day 6 and was particularly exacerbated on day 10 with 326 [16–993] G/L, 95% CI [276–361] in the 90-day survivors group *vs* 198 [11–681] G/L, 95% CI [131–224] in the 90-day non-survivors group (p = 0.0001). In the survivors group, the platelet count returned to the admission value at the end of the first week and continued to rise to become significantly greater than that at admission. In the non-survivors group, the platelet count did not return to the admission values, even after 15 days.

### Kinetics of blood pH and lactic acid

The study of metabolic parameters kinetics revealed that blood pH and serum lactic acid were different between survivors and non-survivors, from the day of admission in ICU to day 4 for lactic acid and to day 6 for blood pH (S1 and S2 Figs). The main differences were observed at day 1 and day 2.

### ROC curves analysis

**Fig 4:**
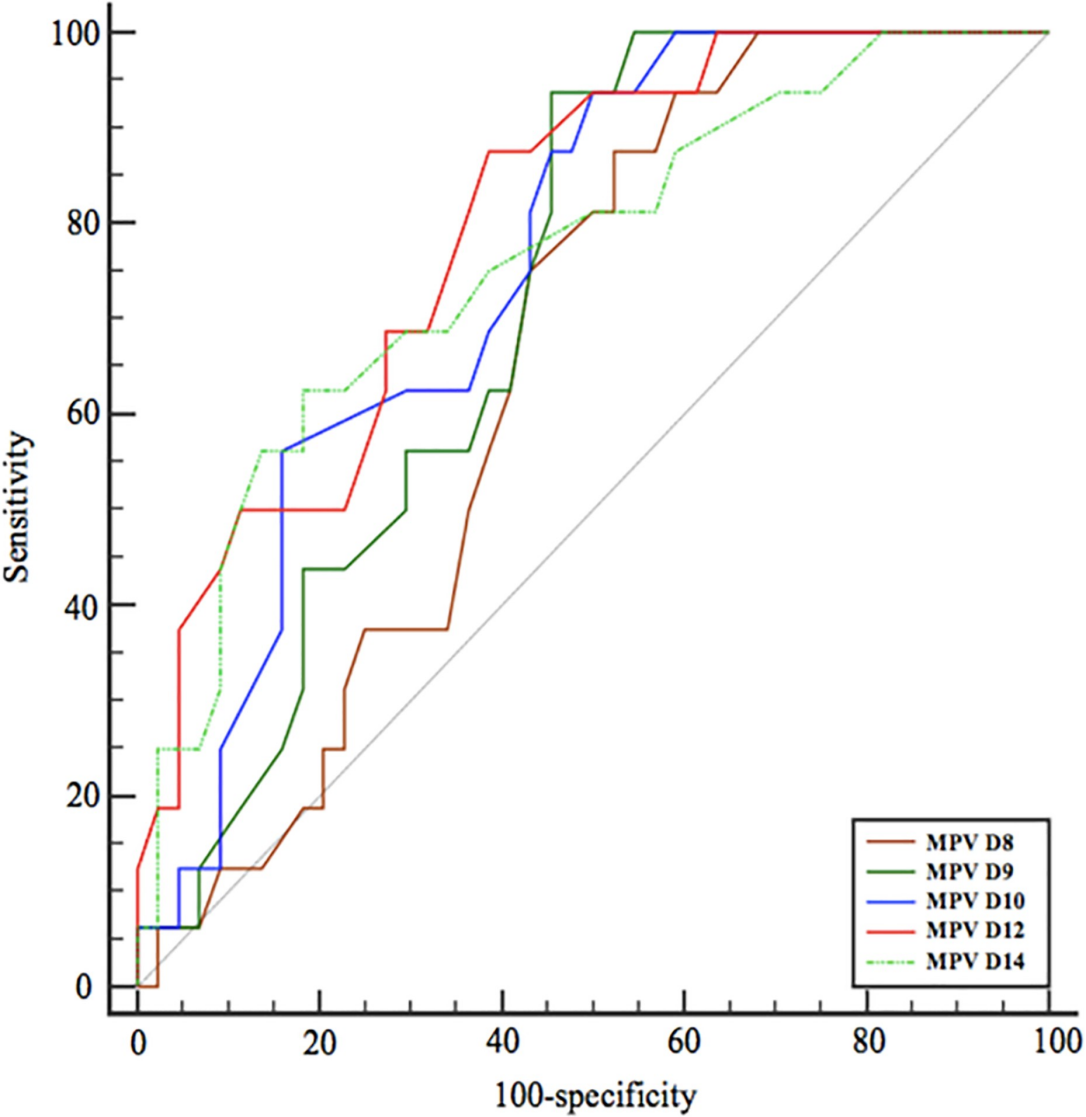
Comparison of areas under ROC curves according to Mean Platelet Volume.

Considering time-to-event, we constructed time-dependent receiver operating characteristics (ROC) curves to assess on which day after the onset of the sepsis MPV provided the better prognostic value for 90-day mortality (**Fig 4**). Areas under the curves (AUC) of the main continuous clinical and biological variates discriminating survivors and non-survivors are shown in **Table 2**. Best MPV value was the 10-day value with a Youden index >11.6fL. Other parameters did not provide acceptable prognostic value in view of AUC or sensitivity/specificity.

**Table 2:** Comparison of area under the curves of continuous clinical and biological variates discriminating survivors and nonsurvivors.

	AUC	95% CI	Threshold	Sensitivity	Specificity	PPV	NPV
**MPV (fL) (Day 10)**	0.76	0.63–0.86	> 11.6	57.45	82.28	49.1	86.7
**SOFA Score**	0.74	0.69–0.79	> 11	73.53	62.81	50.3	82.2
**Ratio MPV/platelet (Day 10) (%)**	0.73	0.59–0.84	> 4.14	72.73	64.08	38.6	88.3
**Base excess (mmol/L) (Day 4)**	0.653	0.58–0.72	≤ 1.9	84.48	41.89	36.3	87.3
**Lactic acid (mmol/L) (Day 0)**	0.614	0.42–0.78	> 5.2	37.11	80.98	50.7	71.0

MPV: Mean Platelet Volume; SOFA: Sequential Organ Failure Assessment; PLT: Platelet; AUC: Area under the curve; CI: Confidence Interval; PPV: positive predictive value, NPV: Negative predictive value

### Kaplan-Meier curve

**Fig 5:**
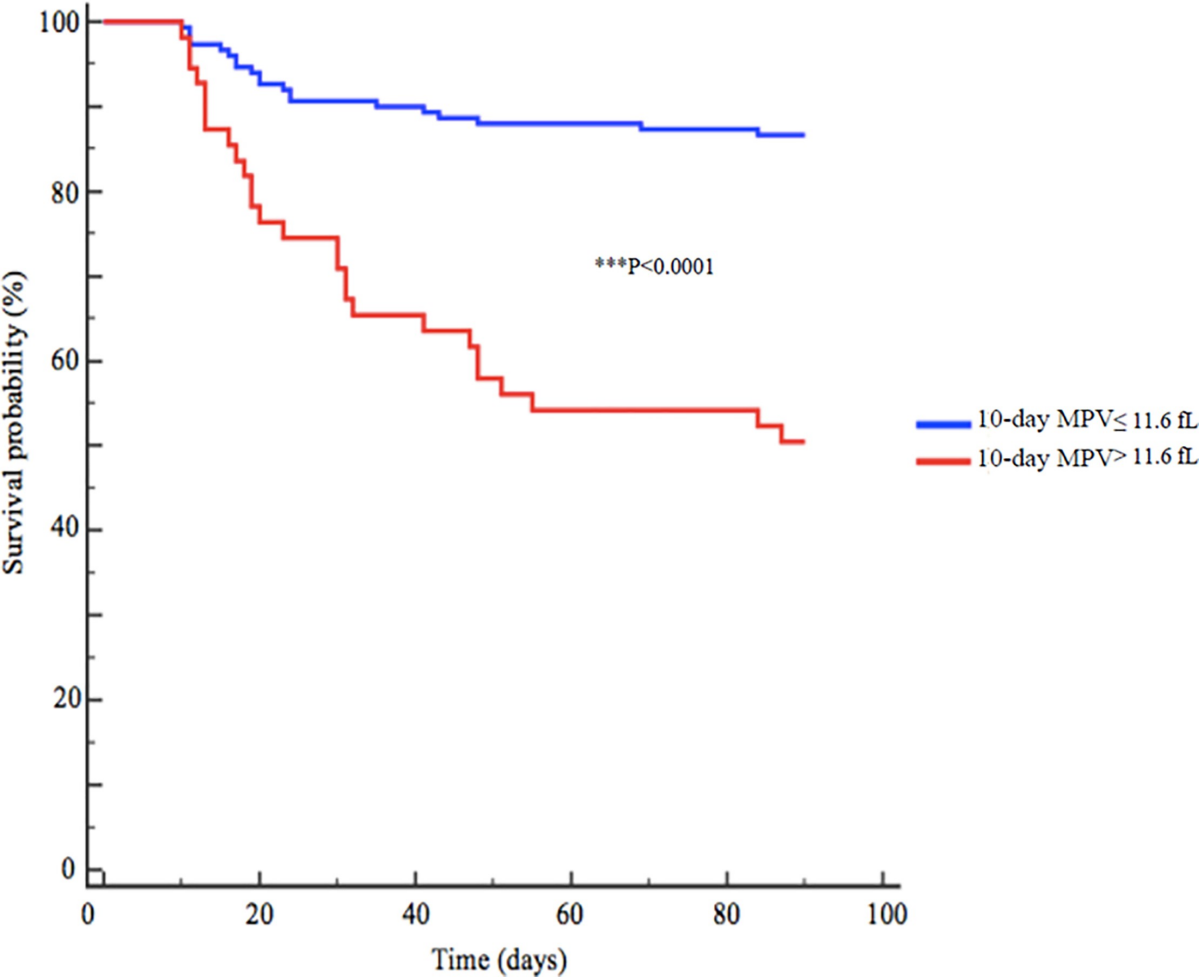
Survival probability Kaplan Meier curve depending on 10-Day MPV.

Kaplan-Meier survival curves were produced using 90-day mortality based on 10-day MPV value >11.6 or ≤ 11.6fL. As shown in **Fig 5**, survival probability was better in patients with 10-day MPV≤11.6fL (p<0.0001) with a survival probability of 86.3%.

### Multivariate analysis

Among the 301 patients included in the study, 102 (33.9%) died within 90 days following admission in ICU. We used multivariate Cox proportional analysis to assess the effect of several covariates on 90-day mortality by adjusting for other significant variables. Covariates included in the multivariate analysis were SOFA score >11, serum lactic acid on admission >5.2 mmol/L, day 4 base excess ≤1.9 mmol/L, 10-day MPV >11.6fL and 10-day/platelet >4.14 (**Table 2**). Cox Model exhibited that 10-day MPV >11.6fL was an independent predictive factor of 90-day mortality (Hazard Ratio (HR) 3.796, 95% CI [1.96–7.35], p = 0.0001) and base excess on day 4 <1.9mmol/L was also a predictive factor of mortality (HR 2.972, 95%CI [1.38–6.40], p = 0.0054 (**Table 3**).

**Table 3:** Multivariate analysis (Cox Model).

Significant Covariates	Hazard-Ratio	95% CI	*P value*
10-day MPV >11.6 fL	3.796	1.96–7.35	0.0001
4-day Base Excess <1.9 mmol/L	2.972	1.38–6.40	0.0054
**Variables not included**			
Renal Replacement Therapy	1.596	0.77–3.29	ns
SOFA > 11	0.957	0.47–1.96	ns

MPV: Mean Platelet Volume; SOFA: Sequential Organ Failure Assessment; CI: Confidence Interval; ns: not significant. Overall model significance with P—0.0001—AUC 0.76 [0.69 to 0.82]

**Fig 6:**
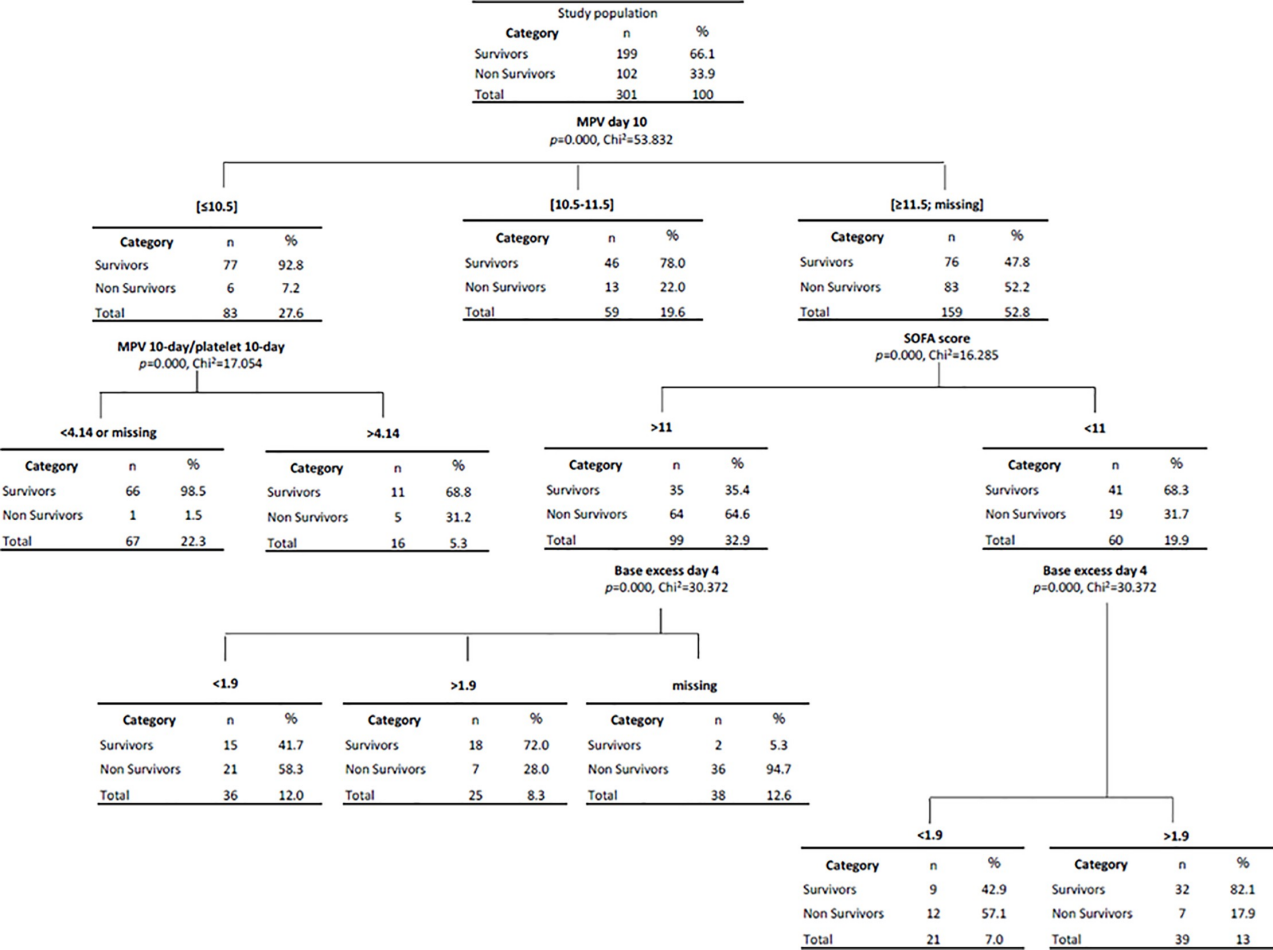
Classification and regression tree (CART) according to 10-day MPV, 10-day MPV/platelet ratio, SOFA score and 4-day base excess.

Results of Classification and Regression Trees (CART) are shown in **Fig 6**. CART have been used extensively as an alternative to the classical linear and additive prediction models. Results are presented in tree form of a decision rule with a hierarchical sequential structure that can be easily understood and applied in clinical practice. The percentage of estimation of this CART analysis was greater than 80%. For example, patients with 10-day MPV ≤10.5fL and MPV/platelet ratio <4.14 had a 90-day predicted survival of 98.5%. This corresponded to 66 patients from the study (21.9%). In contrast, patients with 10-day MPV >11.6fL and with SOFA score >11 at the day of admission presented a 90-day risk of mortality of 64.6%.

## Discussion

Septic shock is a major cause of mortality in ICU; therefore, it is crucial to detect patients at high risk of death in order to improve their management. In this context, the main findings of this study bring new insight that may be useful for the monitoring of septic patients. First, we found that 10-day MPV >11.6 fL was an independent predictive factor of 90-day mortality. Second, we verified the significant increase of MPV during the first 15 days after ICU admission in non-survivors compared to survivors. Third, we confirmed that platelet count in the survivors group, after an early drop, returned to the admission value during the first week whereas it did not in the non-survivors group. The AUC values and multidimensional analysis (COX and CART) showed that MPV was an important factor linked to mortality not sufficiently discriminating if used alone. Other covariates (usually used for mortality prediction in sepsis shock) should be associated, including base excess and lactic acid, as shown by the thresholds of these covariates for the partitioning of patients using CART analysis. This is also the first time that a multidimensional analysis has been completed by a population partitioning in a study on MPV, thus refining the predictability of survival in septic shock with relevant covariates.

Concerning the MPV values, we observed a significant difference from the first day of admission in ICU for septic shock between survivors and non-survivors. Moreover, in the deceased patients group, there was a gradual increase in the MPV values during the first 15 days following the onset of septic shock, whereas, in the survivors group, MPV increased during two days, remained stable until day 7 and then decreased. An increase in MPV value could be associated with uncontrolled infection, as well as linked to illness severity and patient outcome [15,16]. Some studies have highlighted an increase in MPV in infectious endocarditis associated with embolic complications and death while the MPV value decreased in completely healed patients [15,16]. The dosage of MPV and its monitoring during sepsis is relatively simple and would track the evolution of the disease. Several studies have previously demonstrated that the increase in MPV was statistically significant in the first 3 days of gram-positive sepsis[7], could predict 28-day mortality in septic shock [8], and was a risk factor for poor clinical outcome.

Daily monitoring of MPV value would stratify the risk of death in these patients. Special attention should be paid to the evolution of MPV during the first week after the onset of sepsis because the lack of return to its starting value is correlated to an unfavorable outcome. Indeed, septic patients with increased MPV value without a return to the base value are most likely to die and should be tracked in order to improve their management. Some authors propose to use MPV/platelet ratio on admission and at 24 hours to predict mortality at 28 days [10] and that is why this ratio was included in our CART analysis.

The increase in MPV is an important platelet production index that has been shown to correlate with increased platelet reactivity. Beside the physiological platelet production process, previous study has shown that in response to a sharp fall in the platelet count, a rapid production of platelets was possible after megakaryocyte rupture with cytoplasmic fragmentation [13]. Thanks to this alternative mechanism, a large number of platelets is released rapidly into the blood stream with a high proportion of thrombocytes with a large VMP and hence a risk of procoagulant phenotype[16]. As a result, these large platelets are more activated than smaller platelets. An explanation can be that larger platelets, indicating an increased MPV, have more intracellular thromboxane A2 and increased levels of procoagulant surface proteins, such as P-selectin and glycoprotein IIIa, thus presenting a greater prothrombotic potential [17].

MPV is a strong predictive factor of mortality, particularly after day 10. However, rather than the absolute value, kinetics of MPV values appeared to be of major significance, with an absence of decrease in the non-survivors group. Our findings are consistent with a study showing that an increase in MPV after admission to an ICU was independently associated with higher hospital mortality [18]. These findings suggest that progressive MPV increase during sepsis without returning to the initial value is linked to a more severe illness. The trends in changes in MPV and platelets counts are more reliable markers of poor prognosis than the corresponding absolute values.

Platelet count was not different during the first 4 days of the disease between the two groups, and became significantly different at day 6 until the end of the observation (day 15). Platelet count kinetics generally corresponds to a biphasic course that has previously been reported in patients after surgery [19,20] and acute myocardial infarction [21] and could be a physiologic response to stress. In critically ill patients, a similar biphasic pattern has been reported in a study of 18 surgical patients with severe sepsis [22]. Indeed, non-survivors had persistent thrombocytopenia, whereas survival was related to the degree of thrombocytosis within 2 weeks. In critically ill patients, the evolutionary profile of the platelet count is different depending on whether the patient will survive or not. It has even been shown that late thrombocytopenia was more predictive of death than its early onset [22]. In our cohort, the platelet count decreased significantly in the first days after admission to reach a nadir on day 3 in survivors or day 4 in non-survivors. In the survivor group, the platelet count returned to the admission value by the end of the first week and continued to rise to become significantly greater than the admission value by day 8 whereas, in the non-survivors group, platelet count never returned to the admission value during follow-up.

Aydemir et al. have evaluated kinetics of platelet counts and MPV in adult with sepsis to determine whether the responses were specific to the type of infection [7]. They found a day of nadir different between Gram-positive septic patients, gram-negative septic patients and fungal septic patients. They concluded that fungal sepsis had a stronger association with thrombocytopenia and increased MPV. We did not find a difference in platelet counts according to the pathogen agent. This may be explained by the fact that the authors excluded all patients deceased before day 10 or with negative blood cultures contrary to our study. Previous studies have already reported such conflicting results [23,24]; therefore, it is difficult to predict the type of pathogen agent depending on the kinetics of MPV or platelet count.

Mean platelet volume therefore represents a prognostic marker of interest in septic shock and its value is higher in patients who will die. Similar results have previously been shown in other diseases than sepsis. MPV appeared to be as a useful marker for early mortality and neurologic outcomes in patients who achieved return of spontaneous circulation after out-of-hospital cardiac arrest [25]. An elevated MPV was independently associated with increased 30-day mortality, with the highest discriminative value being obtained upon admission after cardiac arrest. An elevated MPV on admission was also associated with poor neurologic outcomes [26]. In another study, MPV was an independent predictor of the risk of stroke among individuals with a history of stroke or transient ischemic attack. Concerning inflammatory diseases like rheumatoid arthritis, MPV has been correlated with inflammatory markers and with the measures of disease activity [26,27].

Other indices of platelets have been described, including platelet volume distribution width (PDW), plateletcrit (PCT), and platelet large cell ratio (PLCR). All these indices can be measured by an inexpensive and readily available routine blood count; however their use and application in septic shock remains unknown [7] and these parameters are not routinely done in our lab, and have not been reported for this study.

The novelty of the study is the use of classification and regression tree (CART) methodology that is a recursive partitioning method for predicting continuous dependent variables (regression) and categorical predictor variables (classification). It is a simple, accurate prediction model for outcome in patients with septic shock easily usable for clinicians [14]. Models are easy to read and interpreted using a flow chart diagram.

Our study presents limitations. The retrospective and monocentric nature of the study can limit the external validity of the results. The variation of standard MPV value between different laboratories is a weak point for the realization of a larger scale study. However, our population was similar to that reported in the literature in terms of age, severity and site of infection [10,28]. 90-day mortality in our study was 33.9% which was comparable with most studies [8,10] and in a recent publication reporting the “third International Consensus definitions for sepsis and septic shock”[1]. We did not have plateletcrit values, at the time of the study that could have been markers of interest.

## Conclusion

Mortality related to sepsis and septic shock remains high. Our work confirms the existence of early biological anomalies such as thrombocytopenia and biological markers of tissue hypoperfusion (pH, base excess, lactic acid). Our results suggest that an increase in MPV value is correlated with mortality. 10-day MPV and 4-day base excess values were the main covariates predicting 90-day survival. Based on these data, we propose a segmentation analysis with 80% predictability of 90-day mortality. This study shows that particular attention must be paid to platelet counts and MPV value variations in septic shock.

## Supporting information

S1 Table Spearman rank correlation between platelet count and MPV. (DOCX)

S2 Table Correlation coefficients between SOFA score at admission and MPV. (DOCX)

S1 Fig Comparison of serum lactic acid kinetics according to 90-day survival. (TIF)

S2 Fig Comparison of blood pH kinetics according to 90-day survival. (TIF)
