# Supplementary material for: Kinetics of mean platelet volume predicts mortality in patients with septic shock
Source: PLoS One. 2019 Oct 17;14(10):e0223553. doi: 10.1371/journal.pone.0223553 (PMC6797099; doi:10.1371/journal.pone.0223553)
Supplement: S1 Table — (DOCX) [file pone.0223553.s001.docx]

|  | **D-1 MPV** | **D0 MPV** | **D1 MPV** | **D2 MPV** | **D3 MPV** | **D4 MPV** | **D5 MPV** | **D6 MPV** | **D7 MPV** | **D8 MPV** | **D9 MPV** | **D10 MPV** | **D11 MPV** | **D13 MPV** | **D15 MPV** |
| --- | --- | --- | --- | --- | --- | --- | --- | --- | --- | --- | --- | --- | --- | --- | --- |
| **D-1 platelets** | -0,291 0,0030 |  |  |  |  |  |  |  |  |  |  |  |  |  |  |
| **D0 platelets** |  | -0,315 <0,0001 |  |  |  |  |  |  |  |  |  |  |  |  |  |
| **D1 platelets** |  |  | -0,347 <0,0001 |  |  |  |  |  |  |  |  |  |  |  |  |
| **D2 platelets** |  |  |  | -0,417 <0,0001 |  |  |  |  |  |  |  |  |  |  |  |
| **D3 platelets** |  |  |  |  | -0,428 <0,0001 |  |  |  |  |  |  |  |  |  |  |
| **D4 platelets** |  |  |  |  |  | -0,384 <0,0001 |  |  |  |  |  |  |  |  |  |
| **D5 platelets** |  |  |  |  |  |  | -0,390 <0,0001 |  |  |  |  |  |  |  |  |
| **D6 platelets** |  |  |  |  |  |  |  | -0,302 0,0001 |  |  |  |  |  |  |  |
| **D7 platelets** |  |  |  |  |  |  |  |  | -0,452 <0,0001 |  |  |  |  |  |  |
| **D8 platelets** |  |  |  |  |  |  |  |  |  | -0,440 <0,0001 |  |  |  |  |  |
| **D9 platelets** |  |  |  |  |  |  |  |  |  |  | -0,484 <0,0001 |  |  |  |  |
| **D10 platelets** |  |  |  |  |  |  |  |  |  |  |  | -0,468 <0,0001 |  |  |  |
| **D11 platelets** |  |  |  |  |  |  |  |  |  |  |  |  | -0,508 <0,0001 |  |  |
| **D13 platelets** |  |  |  |  |  |  |  |  |  |  |  |  |  | -0,481 <0,0001 |  |
| **D15 platelets** |  |  |  |  |  |  |  |  |  |  |  |  |  |  | -0,486 <0,0001 |

**S1 Table**: Spearman rank correlation between platelet count and MPV
