# Supplementary material for: Kinetics of mean platelet volume predicts mortality in patients with septic shock
Source: PLoS One. 2019 Oct 17;14(10):e0223553. doi: 10.1371/journal.pone.0223553 (PMC6797099; doi:10.1371/journal.pone.0223553)
Supplement: S2 Table — (DOCX) [file pone.0223553.s002.docx]

|  | | SOFA score |
| --- | --- | --- |
| D-1 MPV | Correlation coefficient Significance Level P | 0,287 0,0034 |
| D0 MPV | Correlation coefficient Significance Level P | 0,266 <0,0001 |
| D1 MPV | Correlation coefficient Significance Level P | 0,304 <0,0001 |
| D2 MPV | Correlation coefficient Significance Level P | 0,142 0,0346 |
| D3 MPV | Correlation coefficient Significance Level P | 0,162 0,0201 |
| D4 MPV | Correlation coefficient Significance Level P | 0,222 0,0011 |
| D5 MPV | Correlation coefficient Significance Level P | 0,264 0,0003 |
| D6 MPV | Correlation coefficient Significance Level P | 0,253 0,0009 |
| D7 MPV | Correlation coefficient Significance Level P | 0,335 <0,0001 |
| D8 MPV | Correlation coefficient Significance Level P | 0,338 <0,0001 |
| D9 MPV | Correlation coefficient Significance Level P | 0,312 0,0001 |
| D10 MPV | Correlation coefficient Significance Level P | 0,398 <0,0001 |
| D11 MPV | Correlation coefficient Significance Level P | 0,347 <0,0001 |
| D12 MPV | Correlation coefficient Significance Level P | 0,356 0,0001 |
| D13 MPV | Correlation coefficient Significance Level P | 0,375 <0,0001 |
| D14 MPV | Correlation coefficient Significance Level P | 0,317 <0,0001 |
| D15 MPV | Correlation coefficient Significance Level P | 0,486 <0,0001 |

S2 Table: Correlation coefficients between SOFA score at admission AND MPV
