# Supplementary figures and images for: Kinetics of mean platelet volume predicts mortality in patients with septic shock
Source: PLoS One. 2019 Oct 17;14(10):e0223553. doi: 10.1371/journal.pone.0223553 (PMC6797099; doi:10.1371/journal.pone.0223553)

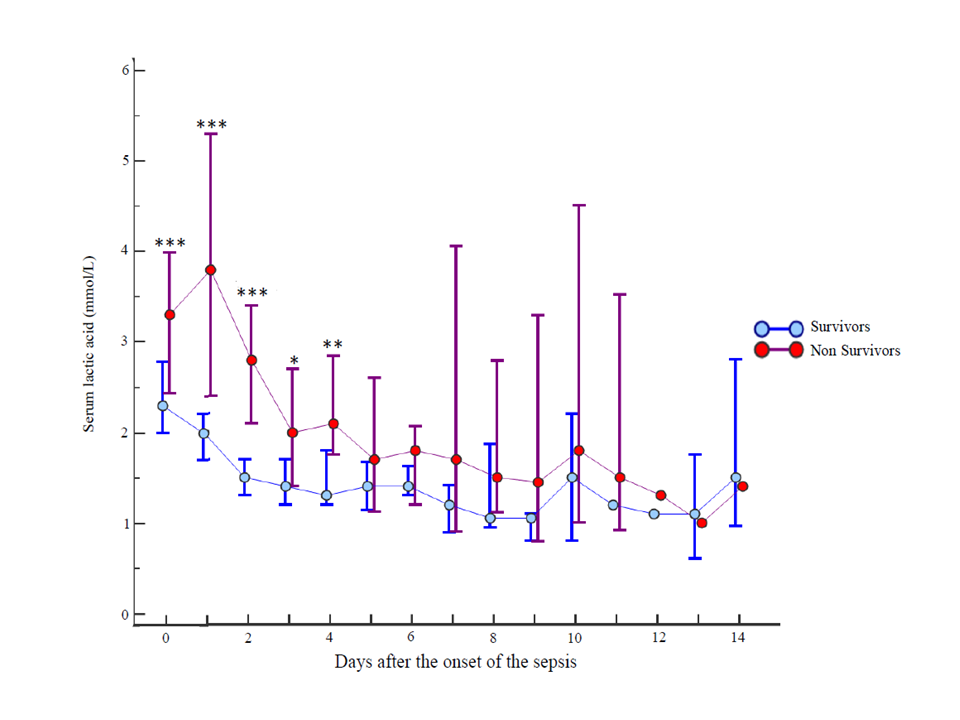

Supplement: S1 Fig — (TIF) [file pone.0223553.s003.tif]

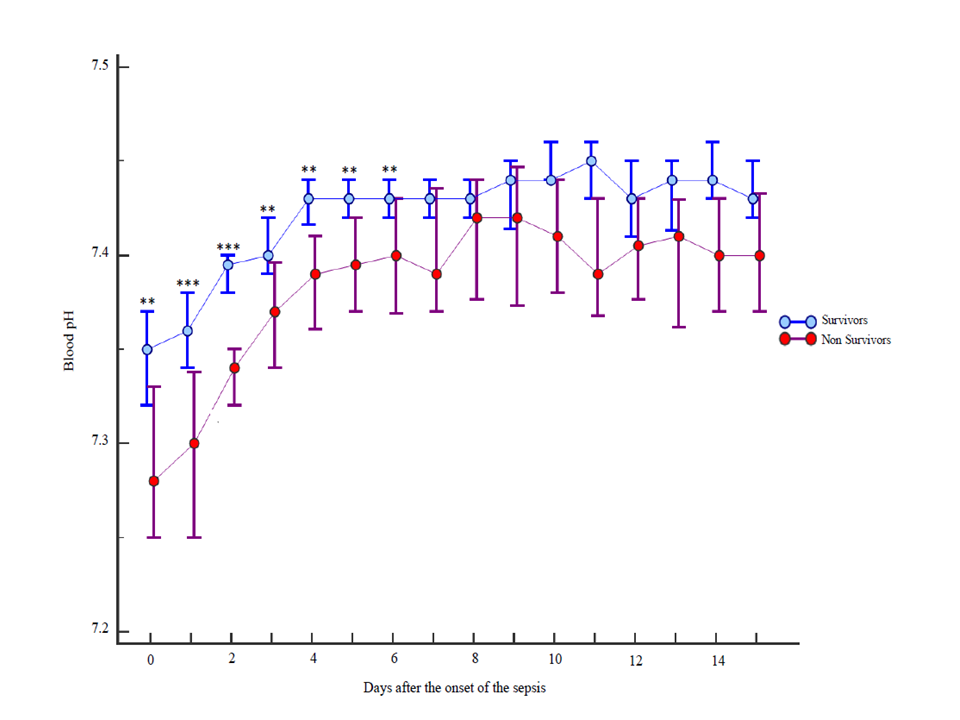

Supplement: S2 Fig — (TIF) [file pone.0223553.s004.tif]
